# Clinical Characteristics, Surgical Management and Outcomes of Sciatic Scoliosis Secondary to Lumbar Disc Herniation: A Systematic Review

**DOI:** 10.3390/life16040589

**Published:** 2026-04-01

**Authors:** Marco Fava, Elena Mendola, Fabrizio Perna, Lavinia Raimondi, Gianluca Giavaresi, Angelo Toscano

**Affiliations:** 1 Ortopedia Generale, IRCCS Istituto Ortopedico Rizzoli, Via di Barbiano, 1/10, 40136 Bologna, Italy; marco.fava@ior.it (M.F.); elena.mendola@ior.it (E.M.); fabrizio.perna@ior.it (F.P.); angelo.toscano@ior.it (A.T.); 2 Scienze e Tecnologie Chirurgiche, IRCCS Istituto Ortopedico Rizzoli, Via di Barbiano, 1/10, 40136 Bologna, Italy; gianluca.giavaresi@ior.it

**Keywords:** scoliosis, sciatica, intervertebral disc displacement, spine, postural deformity

## Abstract

**Background**: Sciatic scoliosis is a nonstructural, compensatory spinal deformity secondary to lumbar disc herniation. In adolescents and young adults, sciatic scoliosis is frequently misdiagnosed as adolescent idiopathic scoliosis due to the low prevalence of lumbar disc herniation in this demographic. Early clinical suspicion is essential, as radiographic features, particularly minimal or absent vertebral rotation on standing radiographs, help distinguish sciatic scoliosis from structural curves such as adolescent idiopathic scoliosis. Key differentiating features include painful scoliosis, a highly positive straight leg raise test, and minimal or absent vertebral rotation on standing radiographs. Delayed diagnosis or inappropriate management may result in residual deformity, highlighting the importance of early surgical decompression. Despite recognition for decades, the literature is fragmented, largely composed of case reports, small series, and retrospective studies, with heterogeneous definitions, radiological assessments, and outcome measures. **Objective**: Provide a comprehensive, up-to-date systematic synthesis of the clinical presentation, radiological characteristics, management strategies, and outcomes of lumbar disc herniation-associated sciatic scoliosis. **Methods**: Thirteen studies evaluating conventional open discectomy and minimally invasive endoscopic procedures (FEID/PELD) were included. Data on demographics, surgical approach, clinical improvement (VAS, ODI, Macnab), and radiographic correction (Cobb angle, trunk list, sagittal alignment) were extracted and synthesized. **Results**: Surgical decompression consistently leads to clinical improvement. Trunk list and coronal deformity were rapidly corrected, with resolution rates ≥ 85% within 6 months across most series. Both open and endoscopic approaches were effective, with minimally invasive techniques offering advantages in tissue preservation and recovery. **Conclusions**: Sciatic Scoliosis is a reversible, nonstructural deformity that responds reliably to surgical decompression. Accurate recognition, particularly in adolescents and young adults, and timely intervention targeting the underlying nerve root compression are critical for optimal outcomes. This review consolidates fragmented evidence, providing a comprehensive synthesis of current knowledge.

## 1. Introduction

Worldwide, the incidence of symptomatic lumbar disc herniation (LDH) is approximately 1% in the general population [1,2,3]. The main symptoms in most patients with LDH include low back pain associated with sciatica and difficulty in walking [4,5]. In a subset of patients, sciatica may be accompanied by coronal and/or sagittal trunk tilt as well as global spinal imbalance. This phenomenon is generally considered a compensatory response to nerve root irritation caused by disc herniation, although the underlying pathophysiological mechanisms have not yet been fully clarified [6].

Sciatic scoliosis (SS), also referred to as sciatic scoliotic list (SSL), trunk list, trunk shift, or lumbosacral list, is defined as a non-structural lateral deformity or tilt of the trunk secondary to nerve root irritation [7,8]. According to the literature, the reported incidence of SS among adult patients with LDH ranges from approximately 13.2% to 17.7%, while in adolescent populations SS appears to be more frequent, with a widely variable reported incidence ranging from 9% to 82% [6,9,10,11]. SS is widely recognized as a non-structural and potentially reversible deformity, which clearly distinguishes it from idiopathic or other structural forms of scoliosis.

From a pathophysiological perspective, sciatic scoliosis is generally considered an antalgic and compensatory posture adopted to reduce nerve root irritation caused by lumbar disc herniation [6]. Rather than representing a fixed structural deformity, SS reflects a pain-related neuromuscular imbalance associated with lumbar nerve root compression. Consequently, the scoliotic posture is typically transient and tends to improve or resolve following adequate relief of radicular pain, either spontaneously or after surgical decompression [12].

Radiographically, SS demonstrates features that clearly distinguish it from structural spinal deformities. Typical findings include relatively small coronal Cobb angles, minimal or absent vertebral rotation, and characteristic curve patterns, often consisting of a short lumbosacral curve associated with a longer compensatory thoracic or thoracolumbar curve [8]. Several studies [7,8,9,10] have also described marked coronal imbalance, commonly assessed by trunk shift relative to the central sacral vertical line, together with sagittal alignment alterations such as lumbar hypolordosis and anterior displacement of the sagittal vertical axis. More recently, classification efforts have focused on coronal curve patterns based on the positional relationship between the spine and the central sacral vertical line, aiming to improve diagnostic accuracy and treatment planning [13].

From a therapeutic standpoint, management of SS is primarily directed at treating the underlying cause of nerve root compression. Conservative treatments such as physical therapy, analgesics, and, in some cases, bracing are occasionally reported in the literature, sometimes following an initial misdiagnosis as idiopathic scoliosis, and may lead to symptom improvement in selected patients; however, lumbar discectomy is frequently advocated to remove the painful stimulus and facilitate restoration of trunk alignment in patients with persistent symptoms [14,15,16,17]. Both open and minimally invasive techniques, including endoscopic approaches, have been reported to result in rapid pain relief and spontaneous correction of the scoliotic posture. Nevertheless, the timeline and completeness of deformity resolution, as well as the impact of different surgical strategies, remain inconsistently reported across studies.

Despite being recognized for decades, the existing literature on SS is fragmented and largely composed of case reports, small case series, and retrospective observational studies, with heterogeneous definitions, radiological assessments, and outcome measures. To date, a comprehensive and up-to-date systematic synthesis focusing on the clinical presentation, radiological characteristics, management strategies, complications, and outcomes of sciatic scoliosis is lacking.

Therefore, the aim of the present systematic review is to summarize the clinical and radiological characteristics of sciatic scoliosis/sciatic scoliotic list (SS) in adolescents, young adults, and adults, to analyze the surgical techniques used for its management, and to evaluate treatment outcomes; in particular, this review is primarily descriptive and prognostic, focusing on the natural course and timing of resolution of SS after lumbar discectomy in patients presenting with SS. By consolidating the available evidence, this review seeks to improve understanding of SS, optimize its management, and reduce the risk of misdiagnosis with structural scoliosis in clinical practice.

## 2. Materials and Methods

This systematic review was conducted in accordance with the Preferred Reporting Items for Systematic Reviews and Meta-Analyses (PRISMA) 2020 statement [18]. The review question was formulated a priori to describe the time course of resolution of sciatic scoliosis/sciatic scoliotic list (SSL) following lumbar discectomy for lumbar disc herniation (LDH).

### 2.1. Information Sources and Search Strategy

A systematic literature search was performed across multiple bibliographic databases, including PubMed/MEDLINE, Embase, Scopus, and Cochrane, with a time span extending from 1 January 2001 to 30 November 2025. The search strategy employed a combination of terms related to LDH and SS, as well as discectomy (e.g., “lumbar disc herniation,” “sciatic scoliosis,” “trunk list,” “trunk shift,” “coronal shift,” “endoscopic discectomy,” “microdiscectomy,” “discectomy”). In addition, reference lists of included studies and relevant reviews were also screened. The complete electronic search strategies are provided in the Supplementary Materials and Methods.

### 2.2. Eligibility Criteria

Eligibility was defined using a PEO framework. Population (P) included adolescents and adults with symptomatic LDH who presented preoperatively with SS (also described as sciatic scoliosis, trunk list, trunk shift, or coronal shift) and underwent lumbar discectomy. Exposure/Condition (E) was the presence of SSL prior to surgery, defined clinically and/or radiographically. Radiographic definitions were accepted when explicitly reported, including Cobb angle thresholds, apical vertebral translation (AVT), the distance between the C7 plumb line and the central sacral vertical line (CSVL–C7PL), or maximal lateral deviation from CSVL (CSVL-max). Outcomes (O) were (i) the primary outcome: time to resolution of SS after discectomy (mean/median or interval-based estimate); and (ii) secondary outcomes: resolution rates at prespecified time points, changes in coronal and sagittal radiographic parameters, clinical outcomes (e.g., VAS, ODI, JOA, Macnab), recurrence, and complications. We included observational studies (prospective or retrospective) and case series reporting postoperative follow-up after discectomy. Case reports, review articles, in vitro, experimental biomechanical, or cadaveric studies were excluded. Studies were excluded if scoliosis was structural (e.g., idiopathic, congenital, degenerative) or due to non-disc etiologies (e.g., tumor, infection, neuromuscular disease), or if postoperative outcomes were not reported.

### 2.3. Study Selection and Data Extraction

All retrieved records were imported into Rayyan reference manager [19] and duplicates were removed. Two reviewers (M.F. and E.M.) independently screened titles/abstracts and then assessed full texts against the eligibility criteria. Disagreements were resolved by discussion and, when necessary, consultation with a third reviewer (L.R.). The study selection process was documented using a PRISMA flow diagram. Two reviewers (M.F. and E.M.) independently extracted data. Extracted items included: study characteristics (author, year of publication, country, design), patient characteristics (sample size, age, sex), LDH level(s), surgical technique (open vs. endoscopic; when applicable PETD/PEID/FEID/PELD), SSL definition and measurement method, follow-up schedule, radiographic and clinical outcomes, recurrence, and complications. For the primary outcome, time-to-resolution estimates (mean/median or interval-based measures) were extracted as reported. All selected studies were managed using Clarivate EndNote Web (2021).

### 2.4. Outcome Definitions and Harmonization

Because definitions of SS and resolution differed across studies, we prespecified harmonization rules. Resolution of SS was defined using the first available criterion in the following hierarchy: (1) AVT ≤ 10 mm, (2) Cobb angle < 10°, (3) CSVL–C7PL < 20 mm, and (4) CSVL-max < 10 mm. Secondary outcomes included resolution proportions at the core follow-up timepoints (immediate/discharge, 1 month, 3 months, 6 months, 12 months, and final follow-up). We distinguished between radiographic parameters used to define SS at baseline and those used to assess postoperative resolution. The prespecified hierarchy was applied only to follow-up outcome assessment. For each study, we also recorded whether postoperative evidence was direct (threshold-based resolution proportion), composite, proxy (mean values suggesting normalization), or not harmonizable to the prespecified hierarchy. When a study did not report a resolution proportion at a given timepoint but reported mean values suggesting normalization, results were flagged as proxy evidence and handled narratively rather than pooled quantitatively.

### 2.5. Study Quality Assessment

Methodological quality was assessed using the NIH Quality Assessment Tool for Case Series Studies. Two reviewers (L.R. and G.G.) performed the assessment independently, and disagreements were resolved through consensus. Each study was rated as Good, Fair, or Poor using a prespecified operational rule based on key items (clear population/case definition, clear intervention description, valid and consistently applied outcome measures, adequate follow-up, and adequately described results). Weighted bar chart was done by using robvis tool [20].

### 2.6. Data Synthesis and Statistical Analysis

The primary outcome (time to resolution of SS) was summarized narratively and tabulated due to heterogeneity in reporting (e.g., mean vs. median vs. interval-censored estimates) and variable follow-up schedules. Where available, the reported time-to-resolution estimates were presented with corresponding measures of dispersion (e.g., IQR, range, or confidence intervals).

## 3. Results

### 3.1. Study Selection

**Figure 1:**
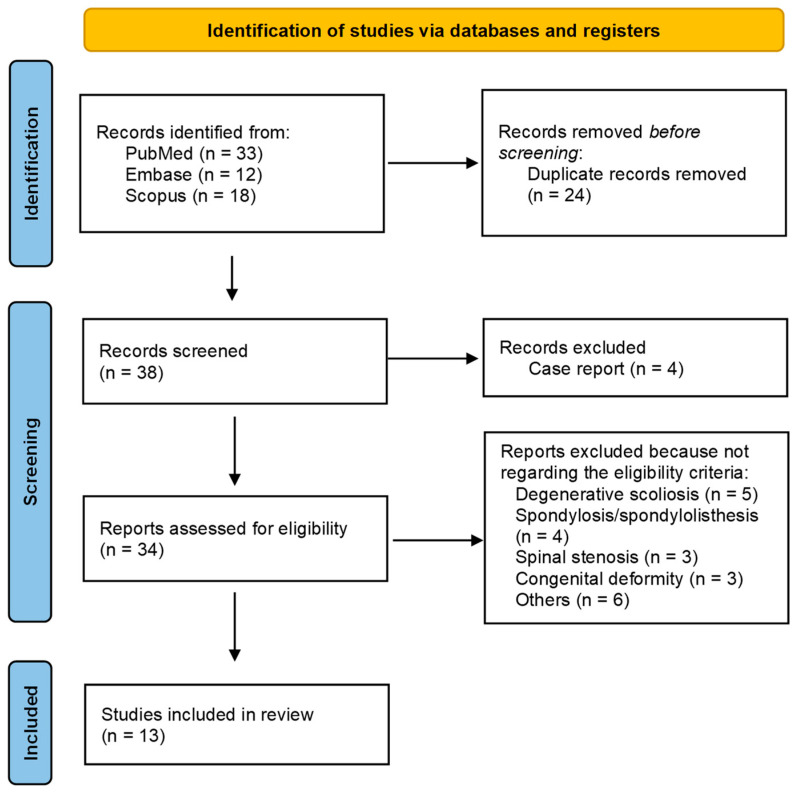
The PRISMA flow diagram illustrates the studies that have been identified, included, excluded.

Following the removal of duplicates, 38 records were screened based on title and abstract. Study screening and selection were performed in accordance with the predefined protocol, and all included studies met the eligibility criteria for evaluating surgical outcomes in patients with LDH associated with non-structural sciatic scoliosis/sciatic scoliotic list or trunk list. Thirty-four full-text articles were assessed for eligibility, and 21 were excluded. The most common reason for exclusion was that they related to other pathologies (e.g., congenital kyphoscoliosis, degenerative scoliosis, spinal stenosis, spondylolisthesis or spondylolisthesis, iatrogenic complications). Other exclusions reflected other criteria (e.g., only radiology assessments, structural scoliosis or non-disc etiologies, or particular populations). Thirteen studies were included in the qualitative synthesis (see PRISMA flow diagram, Figure 1).

### 3.2. Study Characteristics

All included studies reported the preoperative diagnosis, population characteristics, surgical technique, postoperative results, and (when available) complications; extracted data are summarized in Table 1. The evidence base comprised 13 studies published between 2001 and 2025, predominantly retrospective (12/13), with one prospective cohort. Sample sizes ranged from 18 to 134 patients (in some cases reported as SS subcohorts within larger LDH populations). Both adolescents and adults were represented: adolescent cohorts had a mean age of approximately 17–18 years, whereas adult cohorts typically ranged from ~31 to 43 years. Across studies, the most common herniation level was L4–L5, followed by L5–S1, with fewer cases at upper lumbar levels (L1–2 to L3–4).

Overall, the studies evaluated clinical and radiographic outcomes after surgical decompression for symptomatic LDH associated with nonstructural sciatic scoliosis/sciatic scoliotic list (SS/SSL) or trunk list. In some series, nonstructural deformity was explicitly supported by a negative Adams forward bend test and/or by excluding adolescent idiopathic scoliosis (AIS). Interventions included open discectomy/microdiscectomy and minimally invasive endoscopic techniques. Endoscopic approaches utilized are categorized by their surgical route: interlaminar, such as Percutaneous or Full Endoscopic Interlaminar Discectomy (PEID or FEID), and transforaminal, such as Percutaneous Endoscopic Transforaminal Discectomy (PETD). Specialized variants like Unilateral Biportal Endoscopy (UBE) and Microendoscopic Discectomy (MED) provide further technical options. Technique selection was often level-driven (e.g., PEID more commonly used at L5–S1), but the shared therapeutic target across approaches was adequate nerve-root decompression.

Because definitions of SSL and “resolution” varied considerably, postoperative SSL resolution was harmonized using a pre-specified hierarchy: (1) AVT ≤ 10 mm, otherwise (2) Cobb < 10°, otherwise (3) CSVL–C7PL < 20 mm, otherwise (4) CSVL-max < 10 mm. For secondary outcomes, data were preferentially extracted at pre-specified timepoints (immediate/discharge, 1 month, 3 months, 6 months, 12 months, final follow-up). When studies did not provide resolution proportions at those timepoints but reported mean values consistent with normalization, these were treated as proxy evidence and synthesized narratively.

Postoperative coronal outcome assessment was heterogeneous across studies. Direct threshold-based resolution proportions that could be aligned with the prespecified hierarchy were available in 4 studies: AVT in 1 study [9], Cobb angle in 1 study [21], CSVL–C7PL in 1 study [10], and CSVL-max in 1 study [28]. One additional study reported a composite resolution definition based on either C7PL–CSVL < 20 mm or Cobb angle < 10° [22], which was retained narratively because it did not map uniquely to a single hierarchy level. Five studies reported only mean postoperative Cobb angle trajectories suggesting normalization and were therefore treated as proxy evidence rather than pooled proportions [16,23,24,25,26]. One study reported postoperative coronal restoration using trunk shift [27], one reported a scoliosis regression rate without an explicit radiographic threshold [29], and one provided no postoperative resolution outcome [13].

Only a subset of studies provided explicit timing or timepoint-based resolution proportions. Using AVT ≤ 10 mm, most patients achieved resolution within 6 months after open discectomy (85.7% adolescents; 92.7% adults) [9]. In an adolescent microdiscectomy series, “complete resolution” was reported in 83.3% by 6 months and in 100% by the latest follow-up [21]. In a trunk-list cohort using CSVL-max < 10 mm as the normalization threshold, the median time to normalization was 3–6 months and 52% normalized within 12 months after PELD [28]. A FEID cohort reported 68% normalization within 12 months (mean time to improvement ≈ 4 months); because normalization was defined using a composite “OR” criterion (C7PL–CSVL < 20 mm or Cobb angle < 10°), this finding was interpreted as composite normalization rather than a purely Cobb-based estimate, although mean radiographic parameters improved substantially (Cobb 14.0 ± 7.5° to 5.8 ± 5.0°; C7PL–CSVL 36.2 ± 32.5 mm to 15.66 ± 7.11 mm; SVA 40.9 ± 47.8 mm to 19.0 ± 16.2 mm) [22]. In an endoscopic cohort reporting coronal balance acquisition using CSVL–C7PL ≤ 20 mm, the proportion improved from 75–80% at immediate/discharge to 83–89% at 3 months and reached 100% at 6 months [10].

Where longitudinal Cobb angles and/or balance metrics were reported, proxy evidence suggested rapid early improvement followed by continued correction through mid-term follow-up. This pattern was supported by a prospective open discectomy cohort with Cobb reduction documented at 7 days [25] and by a sagittal-imbalance cohort reporting marked improvements in trunk shift and restoration of coronal/sagittal balance on early imaging follow-up [27]. In adolescents undergoing FEID, mean Cobb fell below 10° by 3 months and continued to improve through 6 months and final follow-up, although proportions achieving Cobb < 10° were not reported [23]. Several studies suggested that incomplete radiographic normalization may persist in a small minority (e.g., residual larger curves at final follow-up in a subset) [16] and that double-segment disease may be associated with slower radiographic recovery despite comparable clinical improvement [26].

Across interventional studies, decompression was consistently associated with marked improvements in pain and disability (VAS and, where available, ODI/JOA; one study also reported SF-36 improvement), with high clinical success at final follow-up when reported. Complications and recurrences were generally uncommon and aligned with known discectomy risks; reported events included transient postoperative dysesthesia and occasional recurrence requiring further treatment in some endoscopic cohorts.

Finally, one study reported high “regression rates” at the last follow-up but did not specify resolution thresholds compatible with the pre-specified hierarchy, limiting harmonized interpretation [29]. Another study focused on radiographic characterization/classification and did not report postoperative outcomes; therefore, it did not contribute to time-to-resolution or resolution-rate analyses [13].

### 3.3. Risk of Bias

**Figure 2:**
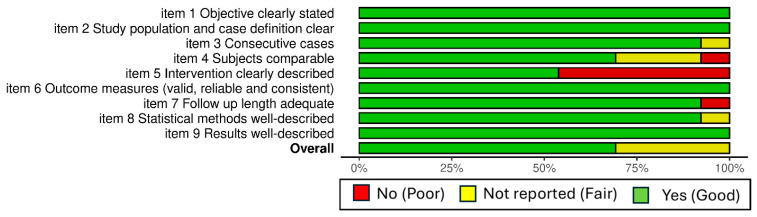
Weighted bar charts of risk of bias assessment of the 13 included studies using the NIH Quality Assessment Tool for Case Series Studies. For each item (1–9) and for the Overall judgement, stacked bars represent the percentage of studies rated Yes, No, or Not reported. In the color legend, the terms in parentheses refer to the Overall rating categories.

The risk of bias was evaluated using the NIH Quality Assessment Tool for Case Series Studies (Figure 2). Most of the studies (9/13, 69.2%) were rated as Good, while the remaining 4/13 (30.8%) were classified as Fair. No studies were rated as Poor. All studies clearly stated the objective and provided a clear study population/case definition (items 1–2:13/13, 100%), used valid and consistently applied outcome measures (item 6: 13/13, 100%), and described results adequately (item 9: 13/13, 100%). The issue of consecutive case inclusion has been addressed in the majority of studies (item 3: 12/13 Yes, 92.3%; 1/13 not reported). Key limitations were identified in relation to comparability of subjects (item 4: 9/13 Yes, 69.2%; 3/13 not reported, 23.1%; 1/13 No, 7.7%) and, most prominently, insufficiently clear intervention descriptions (item 5: 7/13 Yes, 53.8%; 6/13 No, 46.2%). The duration of the follow-up was generally adequate (item 7: 12/13 Yes, 92.3%; 1/13 No, 7.7%), and the statistical methods employed were typically adequately described (item 8: 12/13 Yes, 92.3%; 1/13 not reported, 7.7%).

## 4. Discussion

This systematic review confirms that sciatic scoliosis is a nonstructural, compensatory postural deviation arising from irritation of the spinal nerve root secondary to LDH. Across all surgical techniques analyzed, this postural deviation was consistently highly reversible following effective nerve root decompression.

### 4.1. Incidence and Risk Factors

The prevalence of SS among patients undergoing surgical treatment for LDH is substantial. Adolescents appear to exhibit a higher incidence compared to adults, likely due to greater spinal flexibility and adaptive capacity [9,10,11]. Key risk factors include the anatomical location of the herniation, predominantly at L4–L5, female sex, and severe limitation on the Straight Leg Raise (SLR) test. In particular, the most involved spinal level in patient with LDH and SS was L4–L5 followed by L5–S1. Biomechanical studies suggested that because L4–L5 is not confined to the pelvic cavity, it may be more vulnerable to shift and develop scoliosis compared to L5–S1 [30,31].

### 4.2. Pathomechanism

The lateral trunk shift largely represents a protective, compensatory mechanism: paraspinal muscle spasm and trunk tilt serve to reduce irritation of the affected nerve root. While several studies reported a statistically significant association between the side of disc herniation and the trunk shift [9,10,16] other studies [25,32] found no significant correlation between herniation location (central, paracentral, foraminal) and the apex of scoliosis. Overall, while the direction of trunk list appears to be influenced by the side of sciatica, it cannot be reliably predicted by precise anatomical herniation location.

### 4.3. Efficacy of Surgical Decompression: Endoscopic vs. Open

All surgical approaches analyzed in this review demonstrated high clinical efficacy, characterized by significant reductions in VAS and ODI scores and high levels of patient satisfaction. Conventional open discectomy [7,14,21] provides rapid reversibility of the trunk list, highlighting that nerve decompression is the critical factor for mechanical correction.

Endoscopic techniques [8,20,22,23,25,29,30] offer additional advantages by minimizing soft tissue damage without compromising clinical outcomes. However, a quantitative comparison of perioperative metrics, such as operative time, intraoperative blood loss, and length of hospital stay, was partially limited by inconsistent reporting across the selected literature. In this systematic review, these specific data were explicitly provided by only two studies [22,23], both utilizing endoscopic approaches. In these series, endoscopic discectomy was characterized by negligible blood loss and rapid hospital discharge, typically within 48 to 72 h. Although the lack of systematic reporting in the remaining studies (particularly in open surgery cohorts) precludes a definitive statistical comparison, the available evidence aligns with the known benefits of minimally invasive spine surgery, which may further support the early functional recovery and postural correction of patients with sciatic scoliosis. Finally, it should be noted that more complex cases, such as multilevel procedures, may be associated with a slower radiographic recovery of the spinal alignment [23].

### 4.4. Clinical Implications

In adolescents and young adults, SS is frequently misdiagnosed as idiopathic scoliosis due to the low prevalence of lumbar disc herniation in this population. From a clinical perspective, several practical features may help differentiate these two entities.

First, pain characteristics represent a key distinguishing factor: unlike adolescent idiopathic scoliosis, sciatic scoliosis is typically associated with acute onset lumbar pain and radicular symptoms, often exacerbated by movement and relieved by postural adaptation. Second, neurological findings are frequently present, including a highly positive Straight Leg Raise Test and, in some cases, sensory or motor deficits, which are not expected in idiopathic scoliosis. Third, physical examination typically reveals a negative Adam’s forward bend test, confirming the nonstructural and compensatory nature of the spinal deformity in patients with sciatic scoliosis.

Radiographically, SS is characterized by minimal or absent vertebral rotation despite a visible coronal deviation, whereas structural curves typically show vertebral rotation and more rigid deformity patterns. In addition, SS shows a characteristic curve pattern, often consisting of a short lumbosacral curve associated with a longer compensatory thoracic or thoracolumbar curve. Curve flexibility is another important indicator, as sciatic scoliosis tends to be highly flexible and often corrects in the supine position or after pain relief, reflecting its nonstructural nature.

Failure to recognize these features may lead to inappropriate management, including unnecessary bracing or delayed treatment of the underlying disc herniation. Therefore, careful clinical and radiological assessment is essential to ensure accurate diagnosis and timely intervention. Early surgical decompression, when indicated, plays a crucial role in relieving pain and maximizing the likelihood of spontaneous correction of deformity. The resolution of the scoliotic list is traditionally considered a rapid event following nerve root decompression. However, while the resolution of sciatic scoliosis is a well-established outcome, recent data clarify that complete postural normalization is a gradual process that may extend up to 12 months, providing a clearer prognostic framework for clinicians [22,29]. This is particularly relevant when counseling younger patients who may be concerned about residual trunk asymmetry in the early postoperative weeks.

To further aid clinical decision-making, the key distinguishing features between sciatic scoliosis and adolescent idiopathic scoliosis are summarized in Supplementary Table S1.

### 4.5. Strengths and Limitations

A key strength of this review lies in its methodological alignment with the nature of the available evidence. We adopted a PEO framework rather than PICO/PECO because the aim of this review was primarily descriptive and prognostic, focusing on the natural course and timing of resolution of sciatic scoliosis/sciatic scoliotic list (SSL) after lumbar discectomy in patients presenting with SS. Most eligible studies were single-arm case series or retrospective cohorts reporting postoperative radiographic recovery within the SS group, without a clearly defined or consistently reported comparator group. Therefore, a comparator-based framework (PICO/PECO) would have been misaligned with the available evidence and would have excluded a substantial portion of the relevant literature. In addition, the main outcome of interest—time to resolution—can be addressed without an explicit comparison group, whereas PICO/PECO are more appropriate when the objective is to estimate a causal effect of an intervention or exposure relative to an alternative. For these reasons, PEO provided the most appropriate structure to capture the clinical question and to synthesize the predominantly non-comparative evidence base.

This review is limited by the predominantly retrospective nature of included studies, heterogeneous radiographic definitions, and relatively short follow-up periods, which restrict conclusions regarding long-term maintenance of correction and true recurrence rates. In particular, although a prespecified hierarchy was used to harmonize postoperative resolution across studies, AVT, Cobb angle, and CSVL-based measures are not fully interchangeable, as they capture related but not identical aspects of coronal deformity. Because most studies did not report paired resolution proportions across multiple parameters at the same follow-up timepoint, the extent to which this hierarchy may have influenced classification of “resolution” could not be formally quantified. Standardized radiological criteria and prospective studies with extended follow-up are needed to optimize surgical strategies and confirm these findings.

## 5. Conclusions

SS is a nonstructural, compensatory spinal deformity secondary to lumbar disc herniation. The condition appears to be reversible following effective nerve root decompression, irrespective of surgical technique, age, or curve severity, although this evidence is primarily derived from observational studies. Early diagnosis and timely intervention are critical to prevent the development of residual deformities and maximize functional and radiographic recovery. Surgical management should focus on addressing the underlying pain generator rather than the spinal deformity itself. Although both open and endoscopic approaches are effective, minimally invasive techniques offer advantages in tissue preservation and recovery. Future prospective studies with standardized radiographic criteria and longer follow-up are needed to further define optimal treatment strategies and long-term outcomes.

## Figures and Tables

**Table 1:** Summary of the data extracted from the included studies, which are reported according a prespecified harmonization mapping hierarchy: (1) AVT ≤ 10 mm → (2) Cobb < 10° → (3) CSVL–C7PL < 20 mm → (4) CSVL-max < 10 mm [(C) composite criterion; (P) proxy evidence only; (NH) non-harmonizable/non-standard metric; (NF) no follow-up resolution data].

Ref.	Mapping Hierarchy	Study Design/LOE	SSL Sample (n)/Age (Mean ± SD) (Mean, Range)	LDH Level (n, Patients)	Preoperative Diagnosis	Treatment	Results	Mean Follow-Up Months
Zhang Y et al., 2019 [9]	(1)	Retrospective/III	83 (53 ♂/30 ♀): 42 Adolescents (17.6 ± 2.1); vs. 41 adults (34.5 ± 7.3)	Adolescents: L4–L5 (n = 30); L5–S1 (n = 7); other (n = 5). Adults: L4-L5 (n = 26); L5–S1 (n = 9); other (n = 6)	Resolution defined as AVT ≤ 10 mm; coronal balance as CSVL-C7PL ≤ 20 mm.	Open lumbar discectomy	AVT-resolution within 6 months: 85.7% adolescents, 92.68% adults. Adolescents AVT 46.53 ± 23.54 mm preop → 6.80 ± 3.28 mm at final follow-up; Adults AVT 52.07 ± 24.18 mm preop → 6.87 ± 4.78 mm at final follow-up. Similar curve evolution; age not a prognostic factor.	Adolescents: 28.4 Adults: 28.2
Erdağ & Pehlivanoğlu (2022) [21]	(2)	Retrospective/III	18 Adolescents; 17.1 (14–18)	L4–5 (61%), L5–S1 (39%)	SS/SSL with radiculopathy; negative Adam’s test (non-structural); AIS excluded Cobb < 10° (CSVL–C7PL reported as supportive	Lumbar microdiscectomy	Cobb 25.1° preop → 4.2° final; CSVL–C7PL 4.1 cm → 1.3 cm; “complete resolution” 83.3% at 6 mo, 100% by latest FU	36.8
Pluemvitayaporn T et al., 2025 [22]	(C)	Retrospective/III	60 (34 ♂/26 ♀) Adults (42.9, 19–80)	L3–L4 (n = 8); L4–L5 (n = 34); L5–S1 (n = 18)	Cobb angle > 10° or C7PL–CSVL distance > 20 mm; sciatic scoliotic curve typical short lumbosacral curve mainly three levels (33.33%).	FEID	Normalization of trunk list (C7PL–CSVL < 20 mm or Cobb < 10°) in 68% within 12 months; average time to improvement 4 months.	12
Tu Z et al., 2018 [23]	(P)	Retrospective/III	28 (20 ♂/8 ♀) Scoliotic adolescents (17.8 ± 3.5)	L4–L5 (n = 21); L5–S1 (n = 7)	LDH and sciatic scoliosis; scoliotic group Cobb ≥ 10° (n = 28); comparison group non-scoliotic (n = 46). Radiology: Cobb, CVSL-max, CVSL–C7.	FEID	In scoliotic group: correction achieved immediately after surgery with improvement in VAS leg pain 7.1 ± 1.4 → 1.5 ± 0.9; Cobb 18.4° preop → 8.7° (3 mo) → 5.4° (6 mo) → 2.1° (final FU); CVSL-C7 2.4 ± 0.8 mm → 0.5 ± 0.3 mm at final follow-up. Complications: transient postoperative dysesthesia (n = 1), recurrence (n = 1).	39
Terada Y et al., 2013 [24]		Retrospective/NR	134 (101 ♂/33 ♀) (31, 11–40)	L3/4 (n = 2); L4/5 (n = 58); L5/S1 (n = 73)	Single-level LDH cases (≤40 years, congenital scoliosis excluded); painful scoliosis defined as standing AP L1–L5 Cobb angle ≥ 3°.	Open posterior microdiscectomy/herniotomy without fixation	Sciatic/painful scoliosis present in 77/134 (57%); in 76 evaluable cases, 54/76 (71%) showed convexity on the herniation/ protrusion side. In 36 patients with ≥1-year radiographic follow-up: 3–10° group (n = 33) Cobb 5.3° preop → 2.3° at 1 month → 1.2° at 3–6 months → 1.4° at 1–4 years; >10° group (n = 3) Cobb 20.8° preop → 5.1° at 1 month → 0.9° at 3–6 months → 0.5° at 1–4 years (no between-group difference postoperatively). Two severe cases (>20°) improved early: 24.7° → 0° at 3 months; 27.8° → 4.3° at 4 months.	Subset: 36 patients had ≥12 months follow-up X-ray
Suk KS et al., 2001 [25]		Prospective/IIb	45 (29 ♂/16 ♀) (31.2, 13–62)	L4–L5 (n = 34); L5–S1 (n = 11)	SSL (Cobb angle > 4°).	Open discectomy	Mean Cobb angle preop 9.8° (5–25°); 7 days postop 1.8° (0–14°); mean improvement of symptoms 84.8%; direction of sciatic scoliosis related to side of disc herniation.	Not directly specified (24 months)
Zhu Z et al., 2011 [16]		Retrospective/III	26 (18 ♂/8 ♀) adolescents (17.7 ± 3.3)	L4–L5 (n = 14); L5–S1 (n = 6); L4-L5 and L5-S1 (n = 6)	LDH and sciatic scoliosis; short fractional lumbosacral curves with a long thoracic/thoracolumbar curve toward the opposite side; 21 failed conservative treatment, 5 had pain relief but persistent scoliotic posture.	Open posterior discectomy	Mean Cobb angle of lumbosacral curve: preop 19.5°, postop 11°; proximal curve: preop 24.7°, postop 10.4°; at last follow-up 2 patients had residual lumbosacral curve > 20°; significant association between scoliosis direction and side of disc herniation with trunk shift toward the opposite side.	24
Yang Jitao et al., 2024 [26]		Retrospective/III	74 (33 ♂/31 ♀) (35.2 ± 11.4)	53 Single segment: L4–L5 (n = 25); L5–S1 (n = 20); other single (n = 8). vs. 21 Double segments: L4–L5 and L5–S1 (n = 18); other double (n = 3)	LDH with sciatic scoliosis.	PETD/PEID	Scoliosis corrected spontaneously within 12 months; sagittal curve improved in both groups. Single: VAS leg 7.0 ± 1.2 → 1.3 ± 1.1; Cobb 15.3 ± 2.8° → 5.2 ± 2.3°. Double: VAS leg 6.8 ± 1.5 → 1.2 ± 0.8; Cobb 15.7 ± 4.6° → 2.2 ± 1.0°. Coronal and sagittal balance improvement in double-segment patients may take longer.	18.2
Wang L et al., 2022 [10]	(3)	Retrospective/III	110 (54 ♂/56 ♀) (42 ± 14)	L3/L4 (n = 4); L4/L5 (n = 61); L5/S1 (n = 45)	LDH with spinal imbalance: group A sagittal (n = 31), group B coronal (n = 38), and group C combined (n = 41).	Endoscopic discectomy: PETD n = 39; PEID n = 33; UBED n = 28; MED n = 10)	Group A: 100% acquired sagittal balance immediately; SVA 66.0 ± 19.5 mm → 24.3 ± 9.2 mm. Group B: coronal balance 80% immediately, 89% at 3 months, 100% at 6 months; CSVL-C7PL 31.7 ± 12.8 mm → 13.6 ± 5.7 mm. Group C: sagittal/coronal balance immediately 80%/75%; all sagittal balance by 3–6 months; coronal balance 83% at 3 months, 100% at 6 months; SVA 83.5 ± 40.7 mm → 33.5 ± 17.2 mm; CSVL-C7PL 41.5 ± 22.1 mm → 16.1 ± 7.9 mm.	6
Liang C et al., 2016 [27]	(NH)	Retrospective/NR	25 (17 ♂/8 ♀) (37.4, 25–55)	L1–2 (n = 1); L3–4 (n = 6); L4–L5 (n = 13); L5–S1 (n = 5)	LDH with spinal sagittal imbalance: forward bending posture; C7PL-SVA > 5 cm anteriorly + trunk shift reported	Posterior discectomy via transforaminal percutaneous endoscopic approach (selective discectomy)	All restored coronal and sagittal balance immediately after surgery; C7PL-SVA 11.6 ± 6.6 cm → −0.5 ± 2.6 cm; trunk shift 2.9 ± 6.1 cm → 0.2 ± 0.5 cm; LL 25.3° ± 14.0° → 42.4° ± 10.2°; SS 25.6° ± 9.5° → 30.4° ± 8.7°; TK 24.7° ± 11.3° → 22.0° ± 9.8°; PT 20.7° ± 7.8° → 15.8° ± 5.5° (all *p* < 0.05); ODI 77.8% preop → 4.2% at final follow-up.	28.6
Kim R et al., 2015 [28]	(4)	Retrospective/III	29/164 (10 ♂/19 ♀) (37.1 ± 11.2)	L4–L5 (n = 24); L5–S1 (n = 5)	LDH with trunk list defined as >10 mm shift from midline, normalization CSVL-max < 10 mm	PELD	Trunk list normalized (CSVL-max < 10 mm) in 15/29 (52%) within 12 months; median time to normalization 3–6 months; trunk shift decreased from 36.5 mm (17.7–54.5) to 16.2 mm (10.6–26.9) in patients without normalization.	12
Yang J. et al., 2025 [29]	(NF)	Retrospective/III	76 (45 ♂/31 ♀) (35.4 ± 13.3)	L2–L3 (n = 2); L3–L4 (n = 8); L4–L5 (n = 36); L5–S1 (n = 30)	Patients divided by surgical level: group A L2–3 (n = 2) and L3–4 (n = 8); group B L4–5 (n = 36); group C L5-S1 (n = 30).	PETD (n = 46) PEID (n = 30)	No intraoperative complications. At 1 month: 1 case radiating pain resolved after local block; 1 recurrence treated with open surgery; overall complication rate 3.9%. At last follow-up, scoliosis regression rate: 93.5%, 94.1%, 91.8% in groups A/B/C (no significant difference, *p* > 0.05).	18.6
Zhao Yao et al., 2024 [13]	(NF)	Retrospective/NR	96 with SSL (36.2 ± 8.5) (230 single-segment LDH; 96 SSL; 134 non-SSL; 70 healthy)	SSL group: L4–L5 (n = 60); L5–S1 (n = 36)	SSL defined as Cobb angle ≥ 10° or AVT ≥ 2.0 cm.	PELD	Radiological characteristics in SSL group: Cobb angle 12.5 ± 5.3° (4.2–31.2); trunk shift 26.2 ± 17.9 mm (0–88.2); AVT 31.7 ± 16.0 mm. 62.5% had L4/5 herniation; 76.0% had herniation at the convex side of lumbosacral scoliosis; 81.3% had herniation opposite to trunk shift. Compared with controls: LL and TK decreased, PT increased, SVA moved forward; sagittal imbalance worsened. New coronal classification: Type 1 in 95.8% (91/96).	Not reported (no follow-up data)

Abbreviations: AIS, adolescent idiopathic scoliosis; AP, anteroposterior; AVT, apical vertebral translation; C7PL, C7 plumb line; CSVL, central sacral vertical line; CSVL–C7PL, coronal balance distance between CSVL and C7PL; CSVL-max, maximum coronal deviation from CSVL (maximum trunk shift); FU, follow-up; FEID, full-endoscopic interlaminar discectomy; LDH, lumbar disc herniation; LL, lumbar lordosis; LOE, Level of Evidence; MED, microendoscopic discectomy; NR, not reported; ODI, Oswestry Disability Index; PELD, percutaneous endoscopic lumbar discectomy; PETD, posterior endoscopic transforaminal discectomy; PEID, posterior endoscopic interlaminar discectomy; PT, pelvic tilt; SD, standard deviation; SS, sciatic scoliosis; SSL, sciatic scoliotic list; SVA, sagittal vertical axis; TK, thoracic kyphosis; UBED, unilateral biportal endoscopic discectomy; VAS, visual analog scale.

## Data Availability

No new data were created or analyzed in this study. Data sharing is not applicable.

## Supplementary Materials

The following supporting information can be downloaded at: https://www.mdpi.com/article/10.3390/life16040589/s1, Figure S1: Generic_NIH Case Series traffic light; Supplementary Material and Method: electronic search strategies; Table S1: Differential diagnosis between sciatic scoliosis and adolescent idiopathic scoliosis.

## Abbreviations

The following abbreviations are used in this manuscript: SS, Sciatic scoliosis; AIS, adolescent idiopathic scoliosis; AP, anteroposterior; AVT, apical vertebral translation; C7PL, C7 plumb line; CSVL, central sacral vertical line; CSVL–C7PL, coronal balance distance between CSVL and C7PL; CSVL-max, maximum coronal deviation from CSVL (maximum trunk shift); FU, follow-up; FEID, full-endoscopic interlaminar discectomy; LDH, lumbar disc herniation; LL, lumbar lordosis; LOE, Level of Evidence; MED, microendoscopic discectomy; NR, not reported; ODI, Oswestry Disability Index; PELD, percutaneous endoscopic lumbar discectomy; PETD, posterior endoscopic transforaminal discectomy; PEID, posterior endoscopic interlaminar discectomy; PT, pelvic tilt; SD, standard deviation; SS, sciatic scoliosis; SSL, sciatic scoliotic list; SVA, sagittal vertical axis; TK, thoracic kyphosis; UBED, unilateral biportal endoscopic discectomy; VAS, visual analog scale.
