# Supplementary material for: Clinical Characteristics, Surgical Management and Outcomes of Sciatic Scoliosis Secondary to Lumbar Disc Herniation: A Systematic Review
Source: Life (Basel). 2026 Apr 1;16(4):589. doi: 10.3390/life16040589 (PMC13117475; doi:10.3390/life16040589)
Supplement: Supplementary file 1 [file life-16-00589-s001.zip › Supplementary Material and Method.pdf]

Supplementary Appendix

Systematic Review

Clinical Characteristics, Surgical Management and Outcomes of Sciatic Scoliosis Secondary to Lumbar Disc Herniation: A Systematic Review

Marco Fava[1], Elena Mendola[1], Fabrizio Perna[1], Lavinia Raimondi[2]*, Gianluca Giavaresi[2] and Angelo Toscano[1]

ELECTRONIC SEARCH STRATEGIES

PubMed (MEDLINE)

("sciatic scoliosis"[tiab] OR "antalgic scoliosis"[tiab] OR "trunk list"[tiab] OR
"scoliotic list"[tiab] OR "sciatic list"[tiab] OR "lumbosacral list"[tiab] OR
("trunk"[tiab] AND (shift[tiab] OR list[tiab])) OR
("coronal"[tiab] AND (shift[tiab] OR imbalance[tiab])))
AND
("Intervertebral Disc Displacement"[Mesh] OR "Lumbar Vertebrae"[Mesh] OR
"lumbar disc herniation"[tiab] OR "herniated lumbar disc"[tiab] OR
"intervertebral disc herniation"[tiab] OR "disc prolapse"[tiab] OR LDH[tiab])
AND
(discectomy[tiab] OR decompression[tiab] OR microdiscectomy[tiab] OR
endoscop*[tiab] OR "percutaneous endoscopic"[tiab] OR PELD[tiab] OR PEID[tiab] OR PETD[tiab] OR FEID[tiab])

EMBASE

('sciatic scoliosis':ti,ab OR 'antalgic scoliosis':ti,ab OR 'trunk list':ti,ab OR
'scoliotic list':ti,ab OR 'sciatic list':ti,ab OR 'lumbosacral list':ti,ab OR
(trunk NEAR/3 (shift OR list)):ti,ab OR
(coronal NEAR/3 (shift OR imbalance)):ti,ab)
AND
('lumbar disc herniation'/exp OR 'intervertebral disc herniation'/exp OR
'lumbar disc herniation':ti,ab OR LDH:ti,ab)
AND
('discectomy'/exp OR 'decompression'/exp OR 'endoscopic discectomy'/exp OR
discectomy:ti,ab OR decompression:ti,ab OR microdiscectomy:ti,ab OR
endoscop*:ti,ab OR PELD:ti,ab OR PEID:ti,ab OR PETD:ti,ab OR FEID:ti,ab)

SCOPUS

TITLE-ABS-KEY("sciatic scoliosis" OR "antalgic scoliosis" OR "trunk list" OR "scoliotic list" OR
"sciatic list" OR "lumbosacral list" OR ("trunk" W/3 (shift OR list)) OR
("coronal" W/3 (shift OR imbalance)))
AND TITLE-ABS-KEY("lumbar disc herniation" OR "herniated lumbar disc" OR "intervertebral disc herniation" OR LDH)

AND TITLE-ABS-KEY(discectomy OR decompression OR microdiscectomy OR endoscop* OR PELD OR PEID OR PETD OR FEID)

COCHRANE

#1 ("sciatic scoliosis" OR "antalgic scoliosis" OR "trunk list" OR "scoliotic list" OR "sciatic list" OR "lumbosacral list"):ti,ab,kw
#2 (trunk:ti,ab,kw AND (shift OR list):ti,ab,kw)
#3 (coronal:ti,ab,kw AND (shift OR imbalance):ti,ab,kw)
#4 #1 OR #2 OR #3

#5 MeSH descriptor: [Intervertebral Disc Displacement] explode all trees
#6 MeSH descriptor: [Lumbar Vertebrae] explode all trees
#7 ("lumbar disc herniation" OR "herniated lumbar disc" OR "intervertebral disc herniation" OR "disc prolapse" OR LDH):ti,ab,kw
#8 #5 OR #6 OR #7

#9 (discectomy OR decompression OR microdiscectomy OR endoscop* OR "percutaneous endoscopic" OR PELD OR PEID OR PETD OR FEID):ti,ab,kw

#10 #4 AND #8 AND #9

#11 MeSH descriptor: [Adolescent] explode all trees
#12 MeSH descriptor: [Young Adult] explode all trees
#13 (adolescent* OR "young adult*"):ti,ab,kw
#14 #11 OR #12 OR #13

#15 #10 AND #14
