# Supplementary material for: Clinical Characteristics, Surgical Management and Outcomes of Sciatic Scoliosis Secondary to Lumbar Disc Herniation: A Systematic Review
Source: Life (Basel). 2026 Apr 1;16(4):589. doi: 10.3390/life16040589 (PMC13117475; doi:10.3390/life16040589)
Supplement: Supplementary file 1 [file life-16-00589-s001.zip › Supplementary Table S1.pdf]

Supplementary Table S1. Differential diagnosis between sciatic scoliosis and adolescent idiopathic scoliosis

Feature	Sciatic Scoliosis	Adolescent Idiopathic Scoliosis
**Etiology**	Secondary to lumbar disc herniation (nerve root irritation)	Unknown (idiopathic)
**Pain**	Prominent low back pain with radicular symptoms	Typically painless
**Onset**	Acute or subacute	Gradual, often asymptomatic
**Neurological signs**	Frequently present (positive SLR, possible sensory/motor deficits)	Absent
**Curve pattern**	Nonstructural, often consisting of a short lumbosacral curve associated with a longer compensatory thoracic or thoracolumbar curve	Structural curve
**Vertebral rotation**	Minimal or absent (Nash-Moe grade 0)	Present and progressive
**Curve flexibility**	Highly flexible, negative Adam's forward bend test, improves/corrects in supine position	Less flexible, structural rigidity, positive Adam's forward bend test
**Response to pain relief**	Curve improves with symptom resolution	No change
**Imaging findings**	Disc herniation on MRI	No underlying causative lesion
**Treatment focus**	Treat underlying nerve compression (e.g., discectomy)	Curve monitoring, bracing, or surgery depending on severity
