# Supplementary figures and images for: Clinical Characteristics, Surgical Management and Outcomes of Sciatic Scoliosis Secondary to Lumbar Disc Herniation: A Systematic Review
Source: Life (Basel). 2026 Apr 1;16(4):589. doi: 10.3390/life16040589 (PMC13117475; doi:10.3390/life16040589)

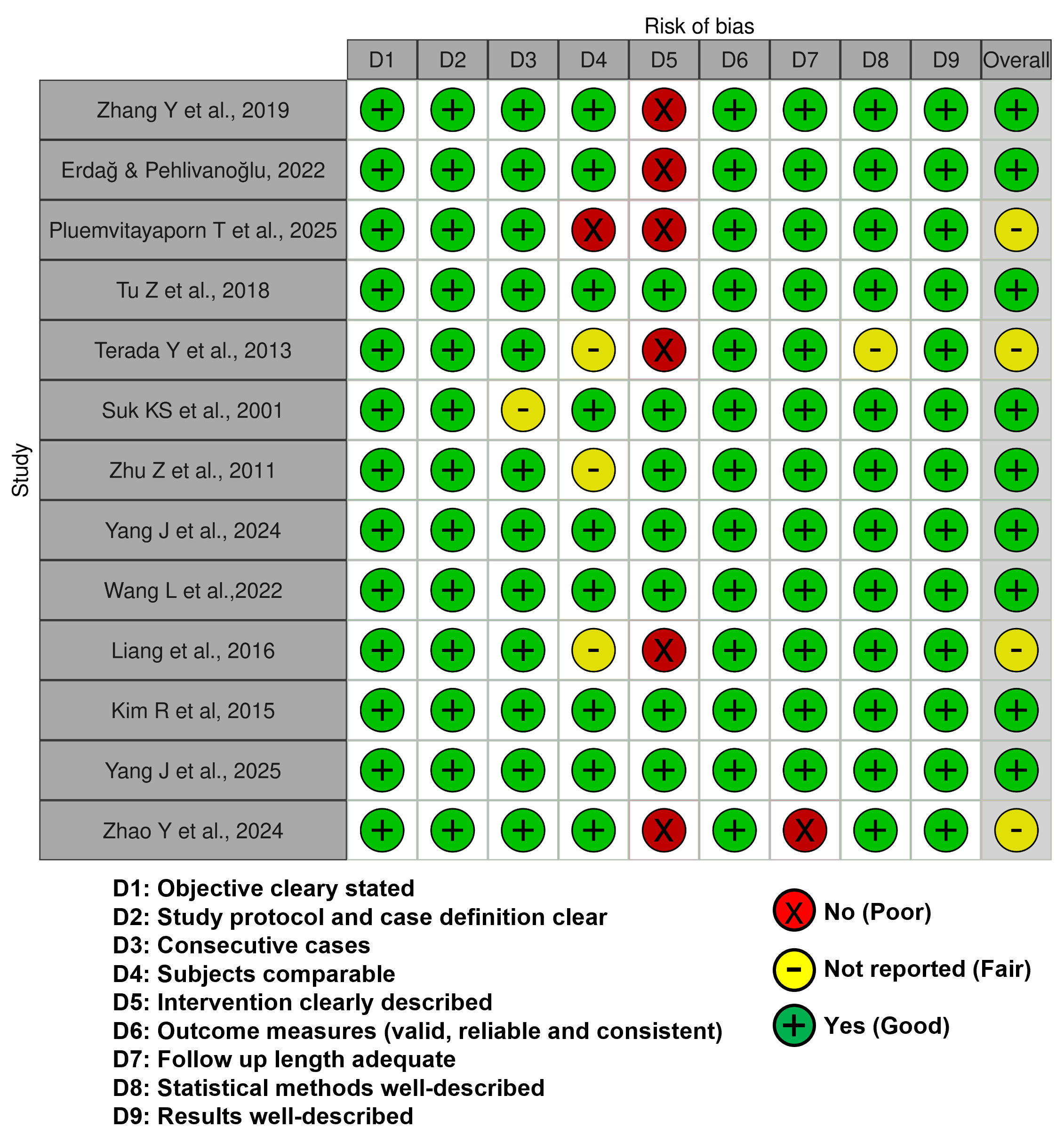

Supplement: Supplementary file 1 [file life-16-00589-s001.zip › Supplementary Figure S1.png]
